# Concepts and Relations in Neurally Inspired *In Situ* Concept-Based Computing

**DOI:** 10.3389/fnbot.2016.00004

**Published:** 2016-05-17

**Authors:** Frank van der Velde

**Affiliations:** ^1^Technical Cognition, CPE-BMS and CTIT, University of Twente, Enschede, Netherlands

**Keywords:** BABI tasks, concepts, computing, *in situ* representations, neural assemblies, reasoning

## Abstract

*In situ* concept-based computing is based on the notion that conceptual representations in the human brain are “*in situ*.” In this way, they are grounded in perception and action. Examples are neuronal assemblies, whose connection structures develop over time and are distributed over different brain areas. *In situ* concepts representations cannot be copied or duplicated because that will disrupt their connection structure, and thus the meaning of these concepts. Higher-level cognitive processes, as found in language and reasoning, can be performed with *in situ* concepts by embedding them in specialized neurally inspired “blackboards.” The interactions between the *in situ* concepts and the blackboards form the basis for *in situ* concept computing architectures. In these architectures, memory (concepts) and processing are interwoven, in contrast with the separation between memory and processing found in Von Neumann architectures. Because the further development of Von Neumann computing (more, faster, yet power limited) is questionable, *in situ* concept computing might be an alternative for concept-based computing. *In situ* concept computing will be illustrated with a recently developed BABI reasoning task. Neurorobotics can play an important role in the development of *in situ* concept computing because of the development of *in situ* concept representations derived in scenarios as needed for reasoning tasks. Neurorobotics would also benefit from power limited and *in situ* concept computing.

## Introduction

Important progress has been made in neurorobotics on topics such as processing sensory information [e.g., Yan et al. (2013) and Chou et al. (2015)], motor control [e.g., Burms et al. (2015) and Grinke et al. (2015)], and implementation with neuromorphic hardware (Stewart et al., 2016). In this way, neurorobotics can use brain research to develop models that process information in a neurally inspired way. Furthermore, the possibility of parallel implementation and neuromorphic hardware may be crucial for further development of robotics, because these forms of hardware can reduce the power of computing while maintaining the ability to process complex information. This allows robots to move around freely without the need for continuous energy take up.

Parallel and neuromorphic forms of hardware [e.g., Benjamin et al. (2014) and Chicca et al. (2014)] are also important given the problems with the further development of standard computer hardware. The past development of Von Neumann computing (more and faster processing, yet power limited) will likely not continue over the next decades (SIA and SRC, 2015). Therefore, new forms of hardware and new computing architectures are needed [e.g., see Nano.gov (2015)]. Williams and DeBenedictis (2015) argue for the development of dedicated “accelerators,” consisting of specific forms of computing that can interact with standard computing to enhance performance on certain tasks. Examples are GPUs for graphical processing. Other examples could be neuromorphic hardware for sensory processing and motor control.

However, robots would also need to develop a form of understanding of the environment they operate in [e.g., Law et al. (2014)]. At some level, they need to acquire concepts of the world around them and use these concepts in basic common sense like reasoning capabilities. However, Davies and Marcus (2015) argued recently that conceptual knowledge and basic forms of common sense reasoning is still lacking in artificial intelligence (AI), and hence also in robotics. In their view, this is even true for a system like IBMs Watson, even though that defeated humans in the game of Jeopardy, which does seem to be concept based.

## BABI Reasoning Tasks

Interestingly, (implicit) support for the view of Davies and Marcus recently arose in the field of machine learning (ML) itself. For example, deep learning has been very successful in topics such as object detection (Krizhevsky et al., 2012), speech recognition (Dahl et al., 2012), and machine translation (Sutskever et al., 2014). But, performance with reasoning is still limited (Bordes et al., 2015; Bottou, 2015). To address this issue, Weston et al. (2015) designed a set of artificial basic reasoning tasks, aptly called “BABI” tasks. There are 20 different BABI tasks (with more to come), which each address a specific form of reasoning. Weston et al. (2015) argue that performing well on all of them is a prerequisite for any system aiming at understanding language and being able to reason.

**Figure 1:**
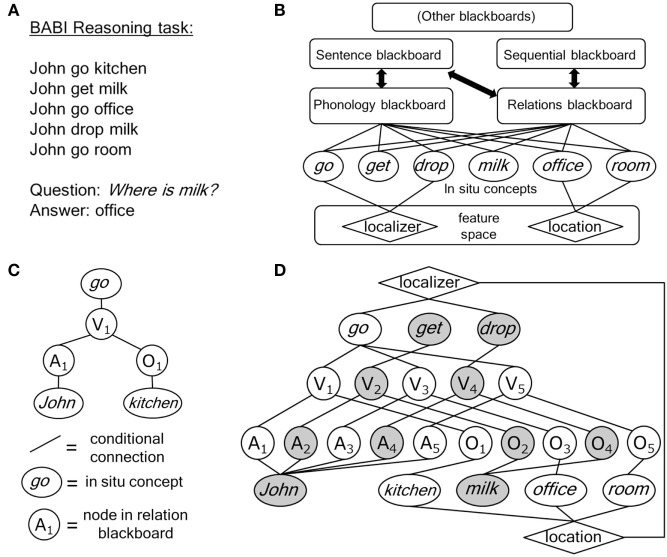
**(A)** An example of a BABI reasoning task [after Bordes et al. (2015)]. **(B)** Architecture for *in situ* concept computing, consisting of a set of identified and not yet identified (“other”) blackboards (ovals represent *in situ* concepts). **(C)** Representation of *John go kitchen* in the *in situ* concept architecture of **(B)** (circles represent nodes in the reasoning blackboard; A, actor; V, verb; O, object). **(D)** Activation (gray nodes and ovals) in the *in situ* concept architecture of **(B)** initiated by the question *Where is milk?*.

Figure 1A illustrates one of the tasks. The sentences represent a simple scenario. The ability of a model to understand the scenario is tested by question answering. To answer the question Where is milk?, a model needs to retrieve two supporting facts. First, John drop milk, which reveals drop as a “localizer” of milk, and then John go office, to retrieve the location. In the BABI tasks, the features and their relations are derived from a simple gaming environment. Concepts are related to features that reveal parts of their meaning. For example, the concept room entails that it is a “location,” and drop entails that an object is “localized” by the action.

The BABI tasks serve as a benchmark for ML. But even if ML is successful on these tasks, there is still the question of whether Von Neumann computing would be suitable as the underlying computing architecture. For example, the concepts in the BABI tasks are derived in gaming scenarios. But, more realistic concepts for language and reasoning will depend on embodied forms of cognition (Parisi, 2010), which, in turn, would require power limited forms of computing (to move around freely). This could be hard to achieve with Von Neumann computing (Williams and DeBenedictis, 2015).

Therefore, it might be that the quest for new forms of computer hardware could coincide with a development of new computing architectures that can also deal with higher-level forms of cognition such as reasoning. Given their relation with higher-level human cognition and the need for new forms of power limited computing (e.g., parallel or neuromorphic computing), these architectures would likely be brain inspired. The BABI tasks could then also be used as a benchmark for these architectures.

Furthermore, instead of using artificial gaming situations, robots interacting with their environment would be ideally suited to develop the scenarios on which the tasks are based and the embodied concepts underlying the reasoning processes in them. Hence, neurorobotics could play an important role in the development and testing of these new computing architectures.

## *In Situ* Concept Computing

Here, I would like to propose and illustrate such an architecture that I refer to as “*in situ* concept(-based)” computing. *In situ* concept computing is brain inspired, because it is based on the notion that in the human brain conceptual representations are “*in situ*.” This means that the information carrying representations (concepts) are not copied or transported but remain where they are, even when they are used in processing and producing complex forms of computing as found in language or reasoning. Examples of such representations are the neuronal assemblies as proposed by Hebb (1949).

Computer architectures based on *in situ* representations provide an alternative for the Von Neumann computer architecture in which a substantial part of the computing time and power derives from moving data over the data bus between memory and processor. Furthermore, *in situ* representations (concepts) provide a direct control on computing (as they do in the brain), which could provide huge benefits for cognitive forms of computing.

BABI tasks and their scenarios derived from neurorobotics could be used as a benchmark for *in situ* concept computing. The best (but not optimally) performing ML models on the tasks (to date) are the memory networks (MemNNs) of Weston et al. (2015). Their performance illustrates the difference between *in situ* concept computing and computing on von Neumann architectures.

A MemNN handles the task in Figure 1A by comparing the question with each of the sentences in the memory. A comparison between two sentences (or between a sentence and a question) is based on the words in the sentences, using additional information. This includes the features that words possess (e.g., <location> is a feature of *room*) and the fact that there is a timing or sequential order of the sentences (e.g., *John go office* occurs after *John get milk* in the scenario). The model compares the feature representations of the two sentences using a comparison function trained on a training set. After training, the model will succeed in the task of Figure 1A if it first selects *John drop milk* with the question *Were is milk?*. Then, the model uses the selected sentence and the question to select a second relevant fact. It will succeed it if selects *John go office*. This produces *office* as the answer to the question. It will select *John go office* instead of *John go kitchen* (and *John go room*) due to the timing difference between these sentences.

The manner in which a MemNN selects the answer to the question illustrates the amount of computing time and power in moving data between memory and processor in a Von Neumann computer architecture. The model needs to compare the question *Were is milk?* with each of the sentences in its memory. This would include irrelevant facts (e.g., *Ann find keys*) if they are also stored in the memory. After selection of the first fact *John drop milk*, search for the second supporting fact can be limited to sentences occurring before the first retrieved fact. But again, all sentences (including irrelevant facts) occurring before this fact need to be investigated.

A key notion of *in situ* concept computing is to use the *in situ* concepts in the question *Where is milk?*, in particular the concept *milk*, to direct the search process. An outline of *in situ* concept computing is given in Figure 1B. The ovals illustrate the *in situ* concept representations. This is a blunt representation, because it suggests that the conceptual representations have nothing in common. However, as neural assemblies they can be (and will be) distributed over the brain, and different concepts could have partially overlapping assembly structures. An illustration of that is given by the feature space, which represent the features that concepts can have and the relations between these features. Here, one can see overlap between concepts. For example, *go* and *drop* are both connected to the feature <localizer>. In fact, this feature is part of the *in situ* (assembly) representation of each of these concepts.

*In situ* representations (just as neuronal assemblies in the brain) cannot be copied by a processor in a computing process. For example, neuronal assemblies, as originally proposed by Hebb (1949), derive their meaning from the connection structure that they possess. This connection structure will develop over time and could be distributed over wide and different brain areas, depending on the meaning involved in the concepts. It will in part consist of the connections that give rise to the activation of the concepts (e.g., based on perception). But, they would also consist of “outgoing” connections resulting in actions derived from the concept involved (van der Velde, 2015). It is difficult to see how this overall connection pattern could be copied and stored elsewhere in the brain. Furthermore, copying just a part of it would disturb or even destroy the content of the concept involved. So, when concepts are a part of more complex processing, such as a reasoning task as illustrated in Figure 1A, they are not copied and transported. Instead, the *in situ* concept representations are dynamically embedded in several “blackboards” in which specific forms of processing occur.

## Neural Blackboards

*In situ* concept computing in general will consist of embedding *in situ* concepts in several specialized blackboards. As illustrated in Figure 1B, these would include a phonology blackboard, to process and represent words in terms of phonemes. This blackboard will interact with the sentence blackboard presented by van der Velde and de Kamps (2006). This blackboard can process and represent sentence structures based on *in situ* concept representations. It can also solve forms of sentence ambiguity in sentence processing by dynamical competitions within this blackboard. An example is given by the difference between the following sentences:

A: The bird found in room died.
B: The bird found in room debris.

The interpretation distinction between A and B depends on the last word. This makes “The bird found in room” ambiguous. In A, “bird” is the subject of “died.” In B, “bird” is the subject of “found.” Humans can handle this ambiguity without problems, switching easily from interpretation A to B (Lakoff, 2015). That is, the interpretation from one sentence to another is achieved during the processing of the sentences. The neural blackboard for sentence structure resolves this ambiguity during the processing of the sentences by a dynamical competition between the neural representations of the sentences involved (van der Velde and de Kamps, 2015).

Another blackboard would be a “relation blackboard,” which can be used for the reasoning processes, as illustrated in Figure 1A. Figure 1C illustrates how this blackboard represents a sentence in Figure 1A. As in the sentence blackboard, sentences are represented with structure nodes in the blackboard that temporarily bind to the concepts. So, in Figure 1C, *John go kitchen* is represented by binding *John* to A_1_, *go* to V_1_, and *kitchen* to O_1_. All connections in the blackboard are conditional, using gating and memory circuits [for details and simulations, see van der Velde and de Kamps (2006)]. A conditional connection is a connection that can be used only when a condition is met. This constitutes a control of activation, which ensures that the connections are not just associative. Conditions depend on the information that is processed (e.g., on word sequence, when sentences or relations are processed) or on memory of binding (e.g., when sentences or relations have been stored in the blackboard). Memory of binding achieves a (temporal) binding between a concept (e.g., *John*) and a structure node (e.g., A_1_) and between structure nodes (e.g., A_1_ and V_1_). Figure 1D illustrates the representation of all sentences of Figure 1A.

The nature of *in situ* concept computing is illustrated by looking at the effect of posing the question *Where is milk?*. This question activates the *in situ* concept *milk*, as illustrated with the gray node in Figure 1D. This concept then directly controls the processing in the blackboards. For example, it will activate O_2_ and O_4_ in the relation blackboard to which it is bound. By controlling the conditional connections, these nodes can then reactivate the rest of the sentence representations. In this way, two sentences are selected: *John drop milk* and *John get milk*. This process illustrates the computational efficiency of *in situ* concept computing. All other sentences stored in the blackboard, including irrelevant ones like *Anne find keys*, are not retrieved from the memory for comparison with the question, because they do not possess an *in situ* concept activated by the question (here, *milk*).

**Figure 2:**
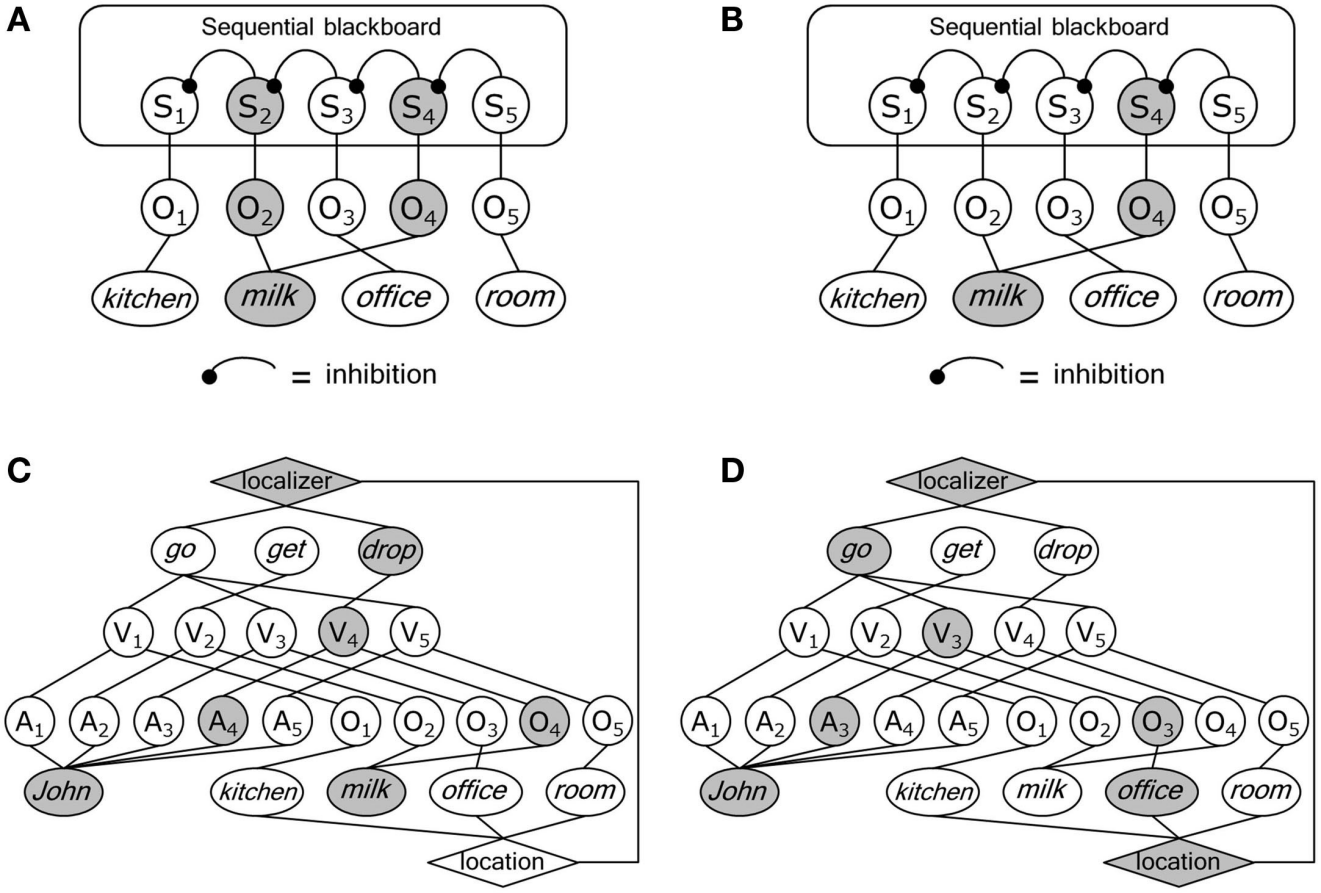
**(A)** Representation of two facts (gray nodes) in the sequence blackboard of Figure 1B bound to the concept *milk* (S, sequence node). **(B)** Selection of the most recent sequence node in the sequence blackboard. **(C)** Selection (gray nodes and ovals) of *John drop milk* in the reasoning blackboard. **(D)** Selection of *John go office* (gray nodes and ovals) in the reasoning blackboard.

In Figure 1D, two sentences in the scenario with the concept *milk* have been selected and one of them has to be eliminated. This can be achieved by the interaction between the relation blackboard and a sequential blackboard, illustrated in Figure 1B. As further illustrated in Figure 2A, the sequential blackboard represents the (temporal) order of the sentences stored in the relation blackboard by binding sequence (S) nodes to the nodes in the relation blackboard. Internal processing in the sequence blackboard can then be used to select an S node based on temporal order. So, O_2_ and O_4_ activate S_2_ and S_4_, respectively, with the latter representing a more recent position in the sequence. As illustrated in Figure 2B, this can be used to deactivate S_2_ (e.g., by inhibition from S_4_, based on the information that the question asks for the most recent position of the *milk*).

Figure 2C illustrates that the selection of S_4_ can initiate the selection of *John drop milk*. Figure 2D illustrates that the second fact in the scenario can be selected by using the information that the question *Where is milk?* asks for a location, and the first selected sentence (*John drop milk*) activates the *in situ* concepts *John* and *drop*. The latter activates the feature <localizer> which is connected to the feature <location>. The combined activation of *John* and <location> initially activates the sentences *John go kitchen* and *John go office* (as occurring before sequential position S_4_). The sentence *John go office* can then be selected as the more recent of the two in the sequence blackboard.

## *In Situ* Concept Computing and Neurorobotics

The introduction of BABI tasks is an important tool to study machine cognition. The difficulties of deep learning with such tasks [e.g., Bordes et al. (2015) and Bottou (2015)] indicate that they require more than discovering statistical regularities. Just finding the relation between a question and a sentence is not enough, because the process needs to be replicated for every step required in the reasoning process. It might be that the information needed to link these steps cannot always be detected in a statistical manner in the source material available.

The problem is aggravated when certain reasoning steps are not given explicitly, but can be assumed on the basis of background or common knowledge (Davies and Marcus, 2015). For example, in the scenario *John get soap*, *John go bathroom*, *John go office*, *Where is soap?*, the step *John drop soap* is omitted after *John go bathroom*. But common sense reasoning would indicate that the soap is in the bathroom.

*In situ* concept computing can be an architecture for an accelerator of conceptual processing and reasoning, as illustrated with the BABI task example discussed above. The architecture as illustrated in Figure 1B is a parallel architecture based on neural principles, which would allow it to be implemented in new forms of (parallel or neuromorphic) hardware.

However, this requires progress on a number of interrelated research lines. Chief among them is the development of concepts, conceptual features, and their relations. The reasoning process in the BABI tasks is influenced by the features that concepts have and the relations between these features. In the case of common sense reasoning, not all information would be directly presented in a given scenario, but instead would be related to background information in the *in situ* conceptual space of the architecture.

As noted, the concept features and their relations in the original BABI tasks (Weston et al., 2015) are set in and derived from a simple gaming environment. For *in situ* concept computing, however, they would have to be grounded in perceptions and actions in realistic environments. This could be achieved by grounding them in the perceptions and actions of a robot behaving in controlled environments. Based on the actions and perceptions of the robot, the connection structures underlying its concepts could develop and integrate in the blackboards, using forms of plasticity (Soltoggio and van der Velde, 2015) that could perhaps be implemented in new forms of hardware (Williams and DeBenedictis, 2015). In this way, conceptual representation and common sense forms of reasoning could be integrated with the perceptual and motor abilities of neurorobotics.

## Development of *In Situ* Concept Computing

*In situ* computing architectures depends on interactions between conceptual domains (e.g., “conceptual spaces”), blackboards, and control circuits, which control the processing dynamics in the architectures. The development of these architectures thus depends on the development of these components and their interactions.

The conceptual spaces in the architectures could be based on existing ontologies, e.g., as derived in robotics [e.g., Prestes et al. (2013)], but they could also be developed by using robots in specific scenarios. The robots could ground concepts and relations between concepts in their perceptions and actions. For example, grounding in robot action sequences could be a basis for *in situ* common sense reasoning that is different from the more linguistic based forms of reasoning. The possibility to do this with robots is an important reason for the integration of *in situ* concept computing with neurorobotics. The “neuro” aspect here derives from the fact that neural representations, as in neuronal assemblies, typically combine grounding with *in situ* forms of representation, which forms the key to *in situ* concept computing.

Next to *in situ* concepts, structured “neural” blackboards are crucial for *in situ* concept computing. Blackboards are also used in computer domains, e.g., to store arbitrary forms of (symbolic) information. The structured blackboards in *in situ* concept computing, however, are fundamentally different. They possess structural information (e.g., related to the structure of relations, as in Figure 1), and they are implemented with dedicated (specialized) structures (e.g., neural circuits, as in the brain). In this way, they cannot store arbitrary information, but they can process information, e.g., by the interactions between the structured representations in the blackboards. As discussed above, pilot simulations (van der Velde and de Kamps, 2015) have shown that this can be used, e.g., for ambiguity resolution in language. Different *in situ* concept computing architectures will be characterized by specialized structured (“neural”) blackboards.

Structured and specialized blackboards also offer new forms of learning. Pilot studies have shown that the distinction between structured blackboards, control circuits, and content addressable activation by *in situ* concepts strongly reduces the number of contingencies that have to be learned (van der Velde and de Kamps, 2010). In this way, learning in *in situ* concept computing architectures is fundamentally different from learning in, e.g., deep learning, which for conceptual processes, as discussed above, depends on Von Neumann forms of computing and repeated searches in large memories for information related to the learning problem at hand [e.g., Weston et al. (2015)].

## Conclusion

Brain inspired forms of computing could play an important role in combining the need for new forms of computing with more sophisticated reasoning capabilities for AI. *In situ* concept computing is an example of brain inspired computing, because it is based on the notion that concept representations are *in situ*, as found with concept representations in the brain. Conceptual forms of processing can be achieved by embedding *in situ* concepts in specialized neurally inspired blackboards. As illustrated with the BABI tasks, *in situ* concepts directly influence processing (without the need to search for them in memory), which reduces the amount of processing and power requirements needed. Neurorobotics could play an important role in developing *in situ* concepts and the scenarios underlying basic and common sense forms of reasoning. Neurorobotics could also benefit from power limited and concept base *in situ* concept computing.

## Author Contributions

The author confirms being the sole contributor of this work and approved it for publication.

## Conflict of Interest Statement

The author declares that the research was conducted in the absence of any commercial or financial relationships that could be construed as a potential conflict of interest.
